# A novel pathway of cGMP

**DOI:** 10.1186/1471-2210-11-S1-O28

**Published:** 2011-08-01

**Authors:** Michaela Kuhn, Beatrice Dankworth, Martin Kruse, Michael Hartmann, Viacheslav O Nikolaev, Katharina Völker, Birgit Gaßner, Robert Feil, Marc Freichel, Olaf Pongs, Michael Klaiber

**Affiliations:** 1 Institute of Physiology, University of Würzburg, Germany; 2 Institut für Neurale Signalverarbeitung, Universität Hamburg, Germany; 3 Institute of Pharmacology, University of Würzburg, Germany; 4 Interfakultäres Institut für Biochemie, Universität Tübingen, Germany; 5 Experimentelle und Klinische Pharmakologie und Toxikologie, Universität des Saarlandes, Hamburg, Germany

## Background

Cardiac atrial natriuretic peptide (ANP) regulates arterial blood pressure, moderates cardiomyocyte growth, and stimulates angiogenesis and metabolism. ANP binds to the transmembrane guanylyl cyclase (GC) receptor, GC-A to exert its diverse functions. This involves a cGMP-dependent signaling pathway preventing pathological [Ca^2+^]_i_ raises in myocytes. In chronic cardiac hypertrophy, however, ANP levels are markedly increased and GC-A/cGMP responses to ANP are blunted due to receptor desensitization.

## Results

Here we show that in this situation ANP binding to GC-A stimulates a novel cGMP-independent signaling pathway in cardiac myocytes, resulting in pathologically elevated intracellular Ca^2+^ levels ([Ca^2+^_i_]). This pathway involves the activation of TRPC3/C6 Ca^2+^ channels (transient receptor potential canonical channel 3/6) by GC-A which forms a stable complex with TRPC3/C6 channels. Our results indicate that the resulting TRPC3/C6-mediated Ca^2+^ entry then stimulates Calmodulin Kinase II (CaMKII) to phosphorylate L-type Ca^2+^ channels leading to increased L-type Ca^2+^ channel mediated Ca^2+^ current and a rise in intracellular Ca^2+^ levels.

## Conclusion

These observations reveal a dual role of the ANP/GC-A signaling pathway in the regulation of cardiac myocyte Ca^2+^_i_-homeostasis. Under physiological conditions, activation of a cGMP-dependent pathway moderates the Ca^2+^_i_-enhancing action of hypertrophic factors such as Angiotensin II. By contrast, a cGMP-independent pathway predominates under pathophysiological conditions, when GC-A is desensitized by high ANP levels. The concomitant rise in [Ca^2+^_i_] is likely to increase the propensity to cardiac hypertrophy and arrhythmias.

